# Migration check tool: automatic plan verification following treatment management systems upgrade and database migration

**DOI:** 10.1120/jacmp.v14i6.4394

**Published:** 2013-11-04

**Authors:** Scott W Hadley, Dale White, Xiaoping Chen, Jean M. Moran, Wayne M. Keranen

**Affiliations:** ^1^ Department of Radiation Oncology Physics The University of Michigan Medical School Ann Arbor Michigan USA

**Keywords:** patient safety, treatment management system, automatic checking

## Abstract

Software upgrades of the treatment management system (TMS) sometimes require that all data be migrated from one version of the database to another. It is necessary to verify that the data are correctly migrated to assure patient safety. It is impossible to verify by hand the thousands of parameters that go into each patient's radiation therapy treatment plan. Repeating pretreatment QA is costly, time‐consuming, and may be inadequate in detecting errors that are introduced during the migration. In this work we investigate the use of an automatic Plan Comparison Tool to verify that plan data have been correctly migrated to a new version of a TMS database from an older version. We developed software to query and compare treatment plans between different versions of the TMS. The same plan in the two TMS systems are translated into an XML schema. A plan comparison module takes the two XML schemas as input and reports any differences in parameters between the two versions of the same plan by applying a schema mapping. A console application is used to query the database to obtain a list of active or in‐preparation plans to be tested. It then runs in batch mode to compare all the plans, and a report of success or failure of the comparison is saved for review. This software tool was used as part of software upgrade and database migration from Varian's Aria 8.9 to Aria 11 TMS. Parameters were compared for 358 treatment plans in 89 minutes. This direct comparison of all plan parameters in the migrated TMS against the previous TMS surpasses current QA methods that relied on repeating pretreatment QA measurements or labor‐intensive and fallible hand comparisons.

PACS numbers: 87.55.T, 87.55.Qr

## I. INTRODUCTION

Modern radiotherapy treatment management systems (TMS) use a large database running on a networked server to provide treatment plan information for delivery at the linear accelerators. The TMS is also used to record the treatment history and other data. The initial treatment plan importation comes under great scrutiny because errors in data transfer can adversely impact patient safety.^(12)^ Pretreatment QA and measurements may be performed to verify the planning data were correctly transferred and are deliverable by a treatment unit.

When a TMS is upgraded to a newer version, a database migration is normally required to map information from the older version of the database schema to the latest schema version. There is significant value in retaining electronic records in the same database for patients who have been previously treated and may require treatment in the future. Maintaining the integrity of the plan information is of the utmost importance to assure accurate and safe treatment after the migration. If one were doing a failure mode and effects analysis of data transfer, then migrating from one database to another would be consider a high‐risk situation with low detectability.^(^ ^3^ ^,^ ^4^ ^)^

In this work we describe a software tool for checking the parameters of a migrated treatment plan against the same plan before migration. The purpose of the tool is to replace a fallible and slow comparison done by users with a comprehensive direct parameter check done by well‐designed software. In a previous upgrade in our clinic from Aria 7.4 to Aria 8.9, we chose to repeat the patient‐specific pretreatment quality assurance measurements for patients under treatment at the time of upgrade to verify the integrity of the treatment plan data. This was approximately a day's worth of effort and included verification of other patient parameters, such as the number of treatments and so on. The tool developed here was used in conjunction with a TMS upgrade and migration from Varian Medical Systems Aria 8.9 to Aria 11 and for a minor upgrade (Aria 11.0.5 to 11 MR1).

## II. MATERIALS AND METHODS

Migration Check Tool (MCT) was developed by our in‐house software development team using Microsoft.NET technology. The primary purpose of the tool is to compare all plans newly imported or under treatment in the database to the same plan in the software upgraded database that contains the migrated data. We refer to the “reference” as the current clinically used database that is to be copied and migrated as part of a software upgrade. The “test” database is the database resulting from the software upgrade and database migration that will go to clinical use after vendor acceptance testing and quality assurance tests have been passed.

### A. System design

**Figure 1:**
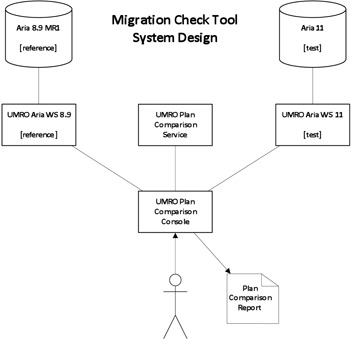
Diagram of software systems, databases, and servers involved in the MCT process.

MCT is a software system comprised of three University of Michigan Radiation Oncology (UMRO) web services and a console application. The web services are part of a larger software architecture and interact with Varian Medical Systems Aria Oncology information system (Varian Medical Systems, Palo Alto, CA) using structured query language (SQL) queries and stored procedure calls. The web services in our system are titled “UMRO Aria WS 8.9”, “UMRO Aria WS 11”, and “UMRO Plan Comparison”. “UMRO Aria WS” are simple access object protocols (SOAP)^(5)^ style web services which provide an interface that exposes key objects stored in the Aria database in XML format. MCT uses each UMRO Aria WS service to retrieve a complete radiotherapy plan object in XML format from the reference database. UMRO Aria WS 11 is exactly the same as the Aria 8.9 version of the service, except that it is adapted to connect to the Aria 11 database. The UMRO Plan Comparison Service is also a SOAP‐style web service, which directly compares two radiotherapy plan objects returning the result of that comparison as an XML document. The use of the SOAP interfaces provides for a platform‐neutral service to provide machine readable information from the Aria database to other applications in need of that information. The UMRO Plan Comparison Console is a Windows Console Application that coordinates use of the three services to compare all plans that are imported or under active treatment in the reference database with the migrated version of each plan found in the test database. Figure 1 is a schematic diagram of the software services and Aria databases. The end user interacts only with the UMRO Plan Comparison Console.

### B. Console Application

The user runs the migration check from the UMRO Plan Comparison Console which provides two distinct features. The first is a feature that searches the reference database to generate a list of active plans to be checked after migration. Active plans are ones that are in an active course and have status of “Unapproved”, “Planning Approved” or “Treatment Approved”. This tool is designed to be run before the TMS database migration to create a list of plans currently in the database that will migrate and likely be used later. This feature allows the users to know ahead of time which plans are targeted for QA during the migration process.

The second feature of the Plan Comparison Console is the batch mode comparison feature. The Plan Comparison Tool takes as input each plan from the two different databases for comparison and runs a comparison algorithm. The active plan list includes the patient ID, course ID, and plan ID, along with other data in XML format. A user can create their own list to include a plan missing from the active plan list or to include plans with other statuses such as “Completed”.

### C. Aria web services

The Aria web service is part of a larger software infrastructure developed at The University of Michigan. The web service translates information in the Aria database into a common XML format for use by other software applications. This XML format with schema mapping is needed because the TMS database schema may be changed in newer versions such that tables cannot be directly compared to one another. For the Aria 8.9 to Aria 11 upgrade, not only did many data tables change, the database server platform also changed from Sybase to Microsoft SQL Server. Use of the common XML format simplifies the comparison algorithm by abstracting the representation of the plan in the database to a common format. Table 1 lists the major radiotherapy parameters that are represented in the XML schema.

**Table 1:** Small sample of parameters queried by Aria Web Service App and the corresponding tolerance limit for parameter comparison

*Field Parameters*	*Value*	*Tolerance*
Field Name	String Compare	Exact Match
Field ID	String Compare	Exact Match
Machine ID	String Compare	Exact Match
Field Type (e.g Static, Dynamic)	String Compare	Exact Match
Monitor Unit	Numeric Value	0.1
Gantry Angle	Numeric Value	0.1 Degrees
Treatment Time	Numeric Value	0.1 Minutes
Field Technique	String Compare	Exact Match
Field Energy	String Compare	Exact Match
Field Mode	String Compare	Exact Match
Dose Rate	Numeric Value	Exact Match
Tolerance Table	String Compare	Exact Match
SSD	Numeric Value	0.1 cm
Collimator Rotation	Numeric Value	0.1 Degrees
Couch Angle	Numeric Value	0.1 Degrees
MLC Control Points	Numeric Value	0.1 cm
Primary Collimators	Numeric Value	0.1 cm
Slotted Field Accessories	String Compare	Exact Match
Other Accessories	String Compare	Exact Match

### D. The Plan Comparison Service

The Plan Comparison Service accepts as input two radiotherapy plan XML documents, one retrieved from UMRO Aria WS 8.9 (reference), and the other from the UMRO Aria WS 11 service (test) by the Plan Comparison Console. The plan from the reference database is considered to be correct, and is the standard to which the migrated plan (test) is compared. This service uses an XML style sheet to make the comparison between the two parsed XML plans using the tolerance levels defined in Table 1. The service generates a report in both XML (for software agents) and PDF (for humans) for each comparison, detailing the parameters that differed between the reference and test plan.

### E. The Plan comparison Tool

**Figure 2:**
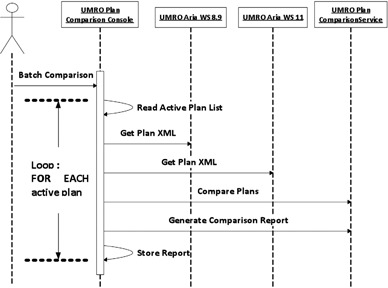
Diagram of MCT process to obtain a list of plans to be checked and the iterative check process.

The Plan Comparison Tool accepts as input the list of plans that are to be compared between the reference and test systems. Figure 2 illustrates the comparison process. The Plan Comparison Tool loops through the list, first getting the plan XML from the Aria WS 8.9, then getting the plan XML from the Aria WS 11, and finally passing both XML documents to the Plan Comparison Service. The Plan Comparison Service compares the two plans, field by field, control point by control point, parameter by parameter (see Table 1), and generates a report that details whether the two plans are identical or, if not, how they differ.

A summary report is generated listing the success or failure of each plan comparison from the active plan list. For each set of plan comparisons, an individual report is generated indicating success or failure.

A risk and hazards analysis of the system was performed to determine the possible deleterious impacts that MCT could have if it is used as part of a database migration and upgrade. A major hazard identified was that plan data would be corrupted but MCT would miss the difference between the test and reference plan. The other major hazard identified was that the wrong databases would be selected for comparison. This could happen in situations where multiple test and development systems exist, such as in our department. To mitigate this hazard, MCT reports which two databases are used in the comparison and that information is verified by physicists and IT staff. In addition, newly imported plans that only exist in the reference database before the migration are noted in the results to verify that the correct databases were used.

MCT was specifically designed to check external beam plans and does not check brachytherapy planning information. By design, the Aria web services do not read or translate information related to images and structures in the Aria database. As such, reference DRRs and CT scans and the structures used for image guidance are not verified by MCT. These data need to be verified by other means. MCT checks the treatment plan geometry and not the dose tracking reference points used to enforce daily, session, and overall dose limits.

### F. Testing and implementation

The Plan Comparison Tool was tested as part of clinical acceptance testing. The software was tested by modifying plans in the reference database with key parameters changed. Plan Comparison Tool was used to compare modified and unmodified plans. The following modifications were made to plans: energy, mode, couch position and angle, deleted IMRT MLC sequence, MLC leaf position for static fields submillimeter changes to MLC leaf position in an IMRT control point, monitor units, jaw positions, gantry and collimator angles, and patient orientation. The tool makes no assumptions with respect to which two fields between two plans should be matched. Instead, it used a measure of agreement calculated by comparing parameters like gantry, couch and collimator angle energy, and treatment mode and treatment type (e.g., static vs. IMRT). The comparison tool has two ways to note a difference between fields. When two fields are matched between two plans, it reports any differences found. If a field is modified a great deal, it will not match any other field and the report will note that no corresponding field was found. Either result is considered a successful detection of a modified field. MCT was tested using clinical Aria 8.9 of VMS Aria against a migrated and upgraded version 11.0.5 of VMS Aria. Tests were run after‐hours, so as not to impact clinical operations.

MCT was implemented during the software upgrade and database migration. Special treatment plans were entered into the database before the upgrade to fully test the available combinations of beam energies and applicators. We labeled these as “one of everything” plans, where fields were included to test every possible combination of X‐ray energy and wedge, electron energy and cone, and various add‐on accessories. The clinical database also includes a large number of test and QA cases for static and IMRT commissioning and other QA applications.

## III. RESULTS & DISCUSSION

MCT was used in conjunction with two separate software upgrades at The University of Michigan Radiation Oncology Department. The first upgrade was from Varáan Medical Systems Aria 8.9 to Aria 11.0.5, which not only involved a database migration but a change in server software from Sybase to Microsoft SQL Server. MCT ran against the clinical database Aria 8.9 at the end of the treatment day to generate an active plan list before the upgrade and migration. A total of 358 plans were listed as active and were tested by MCT after the upgrade. Of the 358 active plans, 212 were test or QA plans and 146 were clinical radiotherapy treatment plans.

A copy of the clinical Aria 8.9 database was set up on an alternate server and activated while the Aria 11.0.5 upgrade and migration was in progress on the clinical server. Once completed, acceptance testing was done and the software was released to our department by the vendor. At that time, a new active plan list was made by running MCT against the Aria 11 database. No differences in the plans listed were found. MCT was then launched in batch mode and each plan in the migrated Aria 11 database was compared to the previous Aria 8.9 database. MCT checked 358 plans in 89 minutes. Reports were generated for each plan tested and no differences were found in any of the plan data. As implemented, the MCT requires two versions of the database to be running concurrently. This may present an obstacle when an additional server is not available to run the previous version of the TMS software and database to compare the migrated system. The use of multiple servers is helpful because it permits a timely investigation of any differences.

Additional database migration testing was done by loading the “one of everything” plans in clinical mode on each treatment unit to verify that the plan parameters were correct and could be loaded by the treatment control software at the treatment unit without errors or warnings. Selected IMRT QA plans that were migrated and tested using MCT were rerun to verify proper IMRT delivery. Each plan that uses CBCT image guidance was tested to verify proper functioning, because MCT does not check image or structure data.

The second use of MCT was for a minor upgrade from Aria 11.0.5 to Aria 11 MR1. Once again, a copy of the clinical database was set up and accessible by SQL queries upon which the web service application relies. In that instance, MCT ran on a virtual machine environment and performance was degraded to where it took nearly 5 hours.

**Figure 3:**
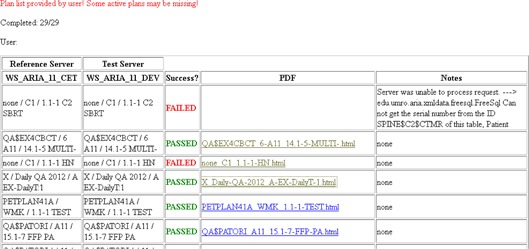
Example of summary report showing passing or failing plan comparison for each plan to be checked.

An example of the output of MCT is show in 3, 4, 5. Figure 3 shows the summary page of all results with links to individual plan comparison reports. In this example, QA cases

Plan list provided by user! Some active plans may be missing!

**Figure 4:**
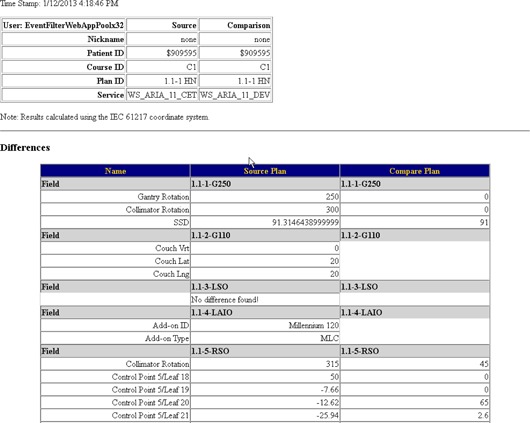
Example report of a plan that was modified to fail plan comparison.

**Figure 5:**
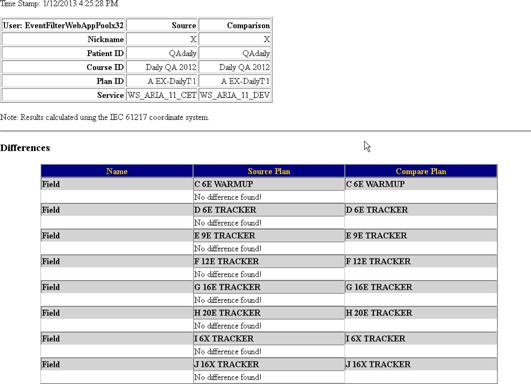
Example report of a plan that had no differences between the pre‐ and postmigrated and updated database.

Completed: 29/29 were modified between the clinical database and test database. The first result, “none / C1 / 1.1–1 C2 SBRT”, demonstrates an instance where a patient doesn't exist in the migrated database. The second comparison that shows a failure, “none / C1 / 1.1–1 HN”, is an example where the plan was artificially modified in Aria to produce differences between the plans. The beginning of this plan comparison report is shown in Fig. 4. The differences are noted in Table 2. MCT reports all differences found and, as a result, the entire report is too long to show here. Figure 5 shows an individual report for a plan that passed the comparison.

Migration Check Tool joins other previous work that used automated software checks of plan parameters at different phases of the radiotherapy planning and delivery process.^(^ ^6^ ^,^ ^7^ ^)^ These computer‐based checks have proven useful in finding errors in process or parameters, and lead to an improvement in patient care. The use of such tools supports patient safety directly and saves invaluable personnel time during the migration process and acceptance.

Many critical parameters that exist in any radiation oncology database are not checked by MCT because it is focused on radiation therapy plan parameters. Items not checked by MCT include, for example, universal IDs, unique serial numbers, patient identifiers, and image data. One critical aspect of treatment not checked by MCT is the dose accumulated to reference points. Reference point data were checked manually by printing a paper report before the migration and then verifying the correct dose after the migration. In our clinic, a physicist verifies the total dose delivered to date for each plan prior to the final migration of the database and then confirms the information in the upgraded system prior to using it for patient treatments. Also, because MCT is only focused on active plans, errors created in plans that have been completed would go undetected. Checking all this type of data would have to be part of a much more comprehensive database integrity check.

**Table 2:** List of artificial plan differences. The example plan used was: “none / C1 / 1.1–1 HN”

*Field ID*	*Change in Field*
1.1–1‐G250	Gantry and collimator angle zeroed out. SSD rounded to whole number.
1.1–2‐G110	Couch position deleted
1.1–4‐LAIO	MLC sequence deleted from field
1.1–5‐RSO	MLC sequence from different field attached.
1.1–6‐RAIO	No changes
1.1–7‐SUP	Primary jaws modified for IMRT field

## IV. CONCLUSIONS

Software upgrades require careful checking of database configuration information stored in the database to assure patient safety and guarantee that the treatment delivery data are correct. The impact of incorrect plan data caused by a faulty migration could be devastating for a patient. With complex IMRT and dynamic arc treatments, it is not possible to manually check all the plan data after a migration. Repeating IMRT QA measurements would be labor‐intensive and even if the repeated measurement passes QA that may not reveal corrupted data used to control treatments.

We developed an automated software check of all treatment plans either under treatment or being prepared for treatment that have been migrated from a clinical version of the TMS to an updated and migrated version. Using a software‐based check of all plan parameters, we were able to make direct comparisons of all parameters used to control complex treatments. The software tool allowed for more parameters to be checked in a more detailed level than users could do manually. This is a vast improvement in quality control compared to manual checks or repeating quality assurance measurements.
